# Polymorphisms in *Myostatin* Gene and Associations with Growth Traits in the Common Carp (*Cyprinus carpio* L.)

**DOI:** 10.3390/ijms131114956

**Published:** 2012-11-14

**Authors:** Yanhong Sun, Xiaomu Yu, Jingou Tong

**Affiliations:** 1 State Key Laboratory of Freshwater Ecology and Biotechnology, Institute of Hydrobiology, Chinese Academy of Sciences, Wuhan 430072, China; E-Mails: sunyanhong0830@126.com (Y.S.); xmyu@ihb.ac.cn (X.Y.); 2 Graduate School of Chinese Academy of Sciences, Beijing 100039, China

**Keywords:** *Myostatin* (*MSTN*), single nucleotide polymorphisms (SNPs), common carp (*Cyprinus carpio* L.), growth traits, association analysis

## Abstract

*Myostatin* (*MSTN*) is a member of the transforming growth factor-β superfamily that negatively regulates skeletal muscle development and growth. In the present study, partial genomic fragments of *MSTN* were screened for single nucleotide polymorphisms (SNPs) in selected common carp individuals from wild populations, and two SNPs in intron 2 (c.371 + 749A > G, c.371 + 781T > C) and two synonymous SNPs in exon 3 (c.42A > G, c.72C > T) were identified. Genotyping by direct sequencing of polymerase chain reaction (PCR) products for these four SNPs were performed in 162 individuals from a commercial hatchery population. Association analysis showed that two SNPs in exon 3 were significantly associated with body weight (*BW*) and condition factor (*K*), and haplotype analyses revealed that haplotype H7H8 showed better growth performance. Our results demonstrated that some of the SNPs in *MSTN* may have positive effects on growth traits and suggested that *MSTN* could be a candidate gene for growth and marker-assisted selection in common carp.

## 1. Introduction

*Myostatin* (*MSTN*), also called GDF-8, belongs to the TGF-β superfamily. It is a growth factor expressed mainly in muscle that regulates development and growth by inhibiting cell cycle progression. McPherron *et al.* (1997) found that *MSTN*-null mice had two to three times more skeletal muscle mass [1]. Due to its role in regulating muscle development and growth, *MSTN* has been considered as an important candidate gene for productivity, growth and performance in domestic animals including pigs [2], sheep [3], chicken [4], rabbits [5], and some aquaculture species such as the bay scallop [6]. A mutation in exon 2 of *MSTN* was significantly associated with growth traits of Zhikong scallop [7]. *MSTN* also potentially represented an important target gene for growth improvement of cultured fish [8].

The common carp (*Cyprinus carpio* L.) is an important freshwater food fish with an annual global production of 3.3 million tons (FAO 2009) [9]. Yu *et al.* (2010) found two SNPs in the *MSTN* of *Cyprinus carpio* var. *jian*, one in exon 1 and the other in intron 2, which were associated with body form and average daily gain [10]. No further studies on *MSTN* polymorphisms and their possible association with growth traits have been reported in common carp.

In the present study, new SNPs were identified in the *MSTN* gene of common carp wild populations. The aim of this study was to further explore the association of *MSTN* polymorphisms with growth traits in an aquacultured common carp population. This study is informative to evaluate *MSTN* as a candidate gene for marker-assisted selection (MAS) for growth in common carp.

## 2. Results and Discussion

Alignment of the common carp *MSTN* fragments from ten unrelated individuals showed two mutations in intron 2 (c.371 + 749A > G, c.371 + 781T > C) and two synonymous mutations in exon 3 (c.42A > G, c.72C > T). The observed and expected heterozygosity ranged from 0.154 to 0.599, and from 0.143 to 0.502, respectively. Two loci (c.371 + 781T > C and c.72C > T) did not significantly deviate from the Hardy-Weinberg equilibrium (*p* > 0.05) (Table 1).

The association analysis between genotypes of single SNP and four growth traits in 162 individuals indicated that the two SNPs in exon 3 were significantly association with body weight (*BW*) and condition factor (*K*). For SNP c.42A > G, the A allele frequency was higher than the G allele frequency (Table 1), and fishes with GG genotype were significantly more associated with *BW* and *K* than fishes with AA and AG (0.001 < *p* < 0.05) genotypes (Table 2). For SNP c.72C > T, the frequency of the C allele was much higher than the frequency of the T allele (Table 1), and fishes with CT genotype had higher *BW* and *K* (0.001 < *p* < 0.05) (Table 2). These results indicated that alleles G and T were associated with a positive effect on BW and K in the tested population of common carp. The two SNPs in intron 2 of the common carp *MSTN* had no significant association with any of the four growth traits.

A total of 13 haplotype combinations were found in the population based on the four SNP loci. Six major haplotype combinations including 141 individuals were used for association analysis, and another seven haplotype combinations were excluded from the association study because each contained no more than seven individuals. For the traits BW and K, individuals with haplotype H7H8 showed better growth performance (*p* < 0.05 or 0.01, Table 3).

Our results showed that the two SNPs in exon 3 of the *MSTN* gene may have a positive impact on the growth traits in common carp. There are two hypotheses to explain how a silent/synonymous variation could have an effect on growth traits. Firstly, a silent/synonymous mutation does not change the amino acid, but it may indirectly affect the function of gene probably in such a way as affecting alternative splicing, splicing efficiency or messenger RNA turnover, and affecting further gene expression. Secondly, a silent/synonymous mutation may be in linkage disequilibrium (LD) with a nearby growth-related quantitative trait loci (QTL). However, a single locus may provide limited information about the association between SNP polymorphisms and economic traits. Some association studies demonstrated that if genotypes of two or more SNPs were linked, they would be more informative [11,12]. Our analyses showed that haplotype H2H2 was associated with poor trait values or growth performance. Consequently, common carp with H2H2 in our study may be abandoned in the selection program. In contrast, fishes with H7H8 showed a much better growth performance, which may have resulted from including the favorable G and T alleles, and those individuals should be kept for further breeding studies. These allele interactions of different SNPs on growth traits in common carp should be further studied. In a previous study, Boman *et al.* (2010) found two mutations that increased muscle mass and reduced fatness in Norwegian white sheep, but only one mutation showed dominance effects [13]. Hickford *et al.* (2010) obtained seven SNPs in Romney sheep *MSTN* not all of which were associated with economically important traits, and they also found that combination analyses among five alleles could provide more information about the association with economically important traits [14].

The advantage of genic SNP markers is their location in DNA regions for coding proteins in the somatotrophic axis, meaning that they are more likely to be near quantitative trait loci (QTLs) which affect growth [15,16]. The use of molecular markers linked to QTLs can provide accurate estimations of breeding values for animals prior to accurate phenotypic information and could be used in MAS [17].

## 3. Experimental Section

Based on the complete sequence for common carp *MSTN* (GenBank accession number: GQ214770.1), a pair of primers (forward: 5′-AGCCTACCATAAAAGGTGTGTG-3′, reverse: 5′-TCAATAGTGTCC ATTCCCAAGT-3′) was designed to amplify a 845 bp fragment of genomic DNA. For SNP discovery, 60 adult common carp were collected from four sites of the Yangtze River system (Dongting Lake, Poyang Lake, Hongze Lake, Shishou section), and ten of these individuals were randomly selected for direct sequencing of *MSTN* fragments for SNP hunting. The obtained sequences included partial intron 2 and total exon 3, corresponding to nt2023–2867 of the GenBank sequence of the GQ214770.1.

For association analysis, a commercial common carp population was generated by crossing 15 males and 15 females, which were hormonally induced during the spawning season in the Zhangdu Lake Fish Farm in Wuhan, China. Juveniles were raised at the same pond until 6 months post-hatching. One hundred and sixty two individuals were randomly sampled, and body weight (*BW*), total length (*TL*) and body length (*BL*) were measured. Condition factor (*K*) was calculated for each individual according to the following formula: *K* = 100 *BW*/*BL*^3^. SNP genotyping was performed by direct sequencing of the PCR products.

Association analyses between genotypes of *MSTN* and growth traits were performed using General Linear Model (GLM) of the SPSS 13.0 software given as: *Y = u + G + e*, where *Y* is the phenotypic value of each trait; *u* is population mean value of four growth traits, *G* is the fixed effects of genotypes of each SNP, and *e* is the random error effect. Popgene32 software was used to test allelic and genotypic frequencies and Hardy-Weinberg equilibrium (HWE), and to calculate observed heterozygosity (*H*o) and expected heterozygosity (*H*e). Haplotypes were obtained for each fish using the software PHASE2.1 [18].

## 4. Conclusion

Four SNPs were identified in common carp *MSTN*, and two SNPs in exon 3 were significantly associated with body weight and conditional factor. Fishes with one of the combined haplotypes (H7H8) also showed better growth performance. This study provides further evidence for the associations of *MSTN* polymorphisms with some growth traits in the common carp, and these results would be valuable to the candidate gene approach and the use of *MSTN* for MAS in common carp or other cyprinid fishes.

## Figures and Tables

**Table 1:** Frequencies of genotypes and alleles for four single nucleotide polymorphisms (SNPs) of *myostatin* (*MSTN)* in the common carp.

Locus	No. of animals	Genotype Frequencies (%)			Allele Frequencies (%)		*H*o	*H*e	HWE
c.371 + 749A > G	162	AA	AG	GG	A	G	0.599	0.502	0.018
		20.37	59.88	19.75	50.31	49.69			

c.371 + 781T > C	162	CC	CT	TT	C	T	0.253	0.231	0.316
		74.07	25.31	0.62	86.73	13.27			

c.42A > G	162	AA	AG	GG	A	G	0.333	0.477	0.0002
		44.44	33.33	22.22	61.11	38.89			

c.72C > T	162	CC	CT		C	T	0.154	0.143	0.599
		84.57	15.43		92.28	7.72			

**Table 2:** Associations between c.42A > G and c.72C > T genotypes of *MSTN* and growth traits in the common carp.

Genotypes	No. of animals	*BW* (g)	*TL* (mm)	*BL* (mm)	*K*
AA	72	144.17 ± 8.32 A	224.72 ± 6.84 a	189.23 ± 5.61 a	2.13 ± 0.13 a
AG	54	144.94 ± 9.05 A	223.61 ± 6.09 a	189.64 ± 5.09 a	2.16 ± 0.11 a
GG	36	150.49 ± 10.87 B	225.44 ± 6.01 a	190.05 ± 5.60 a	2.19 ± 0.12 b
*p*-value		0.003	0.410	0.540	0.047

CC	137	144.74 ± 8.73 A	224.29 ± 6.41 a	189.05 ± 5.38 a	2.14 ± 0.13 a
CT	25	151.77 ± 11.19 B	225.68 ± 6.48 a	190.16 ± 5.74 a	2.21 ± 0.11 b
*p*-value		0.001	0.324	0.350	0.026

A,B The different superscript letters within a column indicate a significant difference (*p* < 0.01);

a,b The different superscript letters within a column indicate a significant difference (*p* < 0.05); The same superscript letter within a column means no significant difference (*p* > 0.05).

**Table 3:** Associations between haplotype combinations of *MSTN* and growth traits in the common carp.

Haplotype combinations	No. of animals	*BW* (g)	*TL* (mm)	*BL* (mm)	*K*
H1H2 ACAC/GCAC	33	145.76 ± 9.66 ab	226.67 ± 7.23 a	190.82 ± 6.05 a	2.10 ± 0.13 A
H1H6 ACAC/GCGC	31	143.79 ± 8.77 a	222.29 ± 6.01 a	187.48 ± 4.88 a	2.18 ± 0.11 AB
H1H7 ACAC/ACGC	18	147.29 ± 10.17 ab	225.00 ± 5.09 a	190.00 ± 5.18^a^	2.15 ± 0.11 AB
H2H2 GCAC/GCAC	23	143.55 ± 7.61 a	221.43 ± 6.77 a	187.17 ± 4.95 a	2.19 ± 0.13 AB
H7H7 ACGC/ACGC	13	146.52 ± 9.75 ab	224.31 ± 5.82 a	189.46 ± 5.59 a	2.16 ± 0.15 AB
H7H8 ACGC/GTGT	23	152.74 ± 11.03 b	226.09 ± 6.15 a	190.39 ± 5.70 a	2.21 ± 0.11 B
*p*-value		0.031	0.235	0.214	0.009

A,B The different superscript letters within a column indicate a significant difference (*p* < 0.01);

a,b The different superscript letters within a column indicate a significant difference (*p* < 0.05); The same superscript letter within a column means no significant difference (*p* > 0.05).
